# Enhancing the detection of barcoded reads in high throughput DNA sequencing data by controlling the false discovery rate

**DOI:** 10.1186/1471-2105-15-264

**Published:** 2014-08-07

**Authors:** Tilo Buschmann, Rong Zhang, Douglas E Brash, Leonid V Bystrykh

**Affiliations:** Max Planck Research Group: Neuroanatomy & Connectivity, Max Planck Institute for Human Cognitive and Brain Sciences, Leipzig, Germany; Department of Therapeutic Radiology, Yale University School of Medicine, New Haven, USA; Laboratory of Ageing Biology and Stem Cells, European Research Institute for the Biology of Ageing, University Medical Center Groningen, University of Groningen, Groningen, The Netherlands; Department of Toxicology, School of Public Health, Hebei Medical University, Shijiazhuang, China; Department of Diagnostics, Fraunhofer Institute for Cell Therapy and Immunology, Leipzig, Germany

## Abstract

**Background:**

DNA barcodes are short unique sequences used to label DNA or RNA-derived samples in multiplexed deep sequencing experiments. During the demultiplexing step, barcodes must be detected and their position identified. In some cases (e.g., with PacBio SMRT), the position of the barcode and DNA context is not well defined. Many reads start inside the genomic insert so that adjacent primers might be missed. The matter is further complicated by coincidental similarities between barcode sequences and reference DNA. Therefore, a robust strategy is required in order to detect barcoded reads and avoid a large number of false positives or negatives.

For mass inference problems such as this one, false discovery rate (FDR) methods are powerful and balanced solutions. Since existing FDR methods cannot be applied to this particular problem, we present an adapted FDR method that is suitable for the detection of barcoded reads as well as suggest possible improvements.

**Results:**

In our analysis, barcode sequences showed high rates of coincidental similarities with the *Mus musculus* reference DNA. This problem became more acute when the length of the barcode sequence decreased and the number of barcodes in the set increased. The method presented in this paper controls the tail area-based false discovery rate to distinguish between barcoded and unbarcoded reads. This method helps to establish the highest acceptable minimal distance between reads and barcode sequences. In a proof of concept experiment we correctly detected barcodes in 83% of the reads with a precision of 89%. Sensitivity improved to 99% at 99% precision when the adjacent primer sequence was incorporated in the analysis. The analysis was further improved using a paired end strategy. Following an analysis of the data for sequence variants induced in the *Atp1a1* gene of C57BL/6 murine melanocytes by ultraviolet light and conferring resistance to ouabain, we found no evidence of cross-contamination of DNA material between samples.

**Conclusion:**

Our method offers a proper quantitative treatment of the problem of detecting barcoded reads in a noisy sequencing environment. It is based on the false discovery rate statistics that allows a proper trade-off between sensitivity and precision to be chosen.

**Electronic supplementary material:**

The online version of this article (doi:10.1186/1471-2105-15-264) contains supplementary material, which is available to authorized users.

## Background

Multiplexed deep sequencing is a cost-saving and time-saving technology used with Next Generation Sequencing that combines and sequences multiple DNA *samples* as one. This method relies on labeling genomic sequences from separate samples with specific tags, also known as *barcodes* [1–4]. These barcodes are short sequences, 3 to 14 nucleotides in length, that are distinct from each other and can have error-correcting properties to protect against the sequence alterations introduced during synthesis, amplification, or sequencing [5, 6]. Recently, two of us proposed the Sequence-Levenshtein distance barcode design that corrects a pre-defined number of insertions, deletions, and substitutions while taking into account any possible DNA sequence that might follow the barcode sequence [7]. It can be easily shown that Sequence-Levenshtein distance-based barcode sets with a minimal distance of 3 or more will correct at least one error.

However, even with the best possible barcode design, recognition of short barcode sequences in the DNA context is often problematic. For example, in many cases the identification of barcode sequences alone cannot be done properly because large genomes provide a full set of all possible combinations of short subsequences (*“words”*) of up to 9 nt length [8], including those reserved for barcodes. Frequencies of words in the genome are neither equal nor random [9] and absent words (also known as *unwords*) could be found for large genomes starting from 10 nt to 11 nt [8]. Curiously, unwords have not received any attention as potential barcodes, whereas small and potentially redundant DNA sequences are largely used instead.

At the moment, the main strategy for recovering short barcodes relies not only on the sequence identity, but also on the expected position of the barcode, which is usually found at the beginning of the sequence either behind a sequencing primer or in front of a PCR primer. This strategy was successfully implemented for Illumina HiSeq machines and Roche Pyrosequencing platforms. For instance, Illumina uses a strategy of separating the barcodes from the analyzed sequence by putting them on different ends of the sequencing adapter [10, 11]. Therefore, the barcode and genomic sequence may be read separately in mutually opposite directions. This approach however is also prone to sequencing errors and barcode misassignments [10, 12]. For example, substitution errors might occur. Also, the beginning of the barcode may be shifted by one or more positions which then appears as an insertion or deletion error in the barcode.

Some newer machines generate longer reads using smaller amount of DNA in the sample. These improvements, however, come with new challenges. The PacBio platform can sequence several kilobases of DNA in one piece [13]. However, the platform is prone to insertion and deletion errors and adds a deliberate time delay before the onset of DNA sequencing, resulting in each DNA molecule having its DNA polymerase positioned at a different location at the start of sequencing [14, 15]. Consequently, the recognition of the barcode position on the basis of primer position alone is imperiled. Theoretically, any sequence can be decoded as a barcode. Therefore a naive decoding of the start of every read would potentially lead to a large number of reads being assigned to the wrong samples or left un-assigned. This decreases the power of the experiment in a multiplexed setup, and cross-contaminates different samples with invalid, precision-decreasing reads. Obviously, such damage to the experimental results is highly undesirable. Thus the detection of barcoded reads in these technologies or in circumstances such as unknown positions of barcodes is an interesting and challenging task.

The problem of detecting the originally attached barcode sequence, the so-called *barcode reference sequences*, in a large number of reads belongs to the category of *large-scale inference problems* (also called *multiple testing problems*) that have been previously successfully approached in statistics using false discovery rate (FDR) methods, for example by Benjamini and Hochberg [16], Efron [17], and Storey [18]. When thousands and millions of statistical hypothesis tests are calculated at the same time, statistically significant results may occur due to random chance (a common problem in GWAS and differential gene expression studies [19–21]). In these FDR methods, the expected proportion of erroneously rejected null hypotheses among the rejected ones is estimated and used as a decision criterion for truly significant results. When some of the hypotheses are indeed incorrectly rejected, FDR methods potentially offer a higher sensitivity than naive or Family Wise Error correction methods that estimate the probability of one or more false discoveries instead [20].

A common approach of FDR methods is to estimate the parameters and shapes of the distributions of null and alternative hypotheses. From the estimated cumulative distribution function, the false discovery rate (1 - precision) is inferred to determine the level of confidence in the significance of an alternative test. This FDR variant is commonly called *tail area-based FDR* and is shortened to *“Fdr”* to distinguish it from other FDR variants [22]. A similar strategy can be applied to the problem of detecting barcoded reads. Every comparison of a read to the experimental barcode set is a statistical test that determines whether the read is barcoded or not. Applying the test to thousands of reads inevitably results in many false detections due to random chance and naturally occurring similar DNA or RNA sequences, so that FDR methods are applicable. However, directly applying the FDR methods mentioned above is not possible, as the distributions of similarities between reads and barcode sets do not follow the assumptions required by these methods. For example, Efron’s method requires a normal distribution of z-values [17], and Storey’s solution requires a uniform distribution of p-values under the null hypothesis [18]. Both methods require that the majority of tests (>80*%*) belong to the null hypotheses, while no such prerequisite can be made for the detection of barcoded reads.

Therefore, our goal was to develop a solution tailored towards making a particular distinction between reads that still contain the attached barcode sequence (“*barcoded reads*”), and reads that start with or within the genomic insert (which we will call “*orphaned reads*” because they are no longer assignable to their original samples). The method provides a way to estimate and control the Fdr of the detection of barcoded reads. Detection of barcoded reads is only the first step in the demultiplexing pipeline, so we further investigated the quality of correcting errors in the detected barcode sequence and thus assigning reads to their original samples.

Our method is intended to be applicable to different sequencing technologies. For demonstration purposes we have tested and investigated the application of the method to a particular technology, which in our case is PacBio SMRT with continuous long reads (CLR).

## Approach

Calculation of the minimal distance *δ* between a read and a set of reference barcodes is a statistical hypothesis test and *δ* is its test statistic. A high minimal distance corresponds to a low likelihood for the read to start with a barcode (the null hypothesis), while a low minimal distance corresponds to a high likelihood of the read to start with a barcode (the alternative hypothesis). Detecting barcodes in a huge number of experimental reads is a form of *multiple hypothesis testing* where a high rate of false detections (Type I errors) is expected. Our approach is to *estimate* and *control* the tail area-based *Fdr* from the frequency function of minimal distances *δ* of the whole empirical set of reads.

The frequency of minimal distances *δ* between barcode reference sequences and reads follows a discrete irregular distribution. This empirical distribution is the mixture of two entities: 1) The distribution of orphaned reads *f*_*orphaned*_(*δ*) (the *null hypothesis* distribution *f*_0_(*δ*)) and 2) the distribution of barcoded reads *f*_*barcoded*_(*δ*) (the *alternative hypothesis* distribution *f*_1_(*δ*)). Estimating the shape and mixing proportions of these two distributions allows us to calculate the Fdr and sensitivity from the estimated cumulative distribution functions  and  when using a distance *δ*_*t*_ as threshold to distinguish barcoded reads (*δ*≤*δ*_*t*_) from orphaned reads (*δ*>*δ*_*t*_). We estimate these two sub-distributions by fitting a set of simulated barcoded and orphaned reads to empirical data. In our model, six parameters influence the simulated distributions: the fraction of barcoded reads, the sequencing error rate, the variance of the sequencing error rate and the ratios of insertions, deletions, and substitutions. For parameter search, we decided to use an evolutionary algorithm which finds a set of parameters that best fits the simulated data to the empirical data. Details of this algorithm are presented in the Methods section.

**Figure 1:**
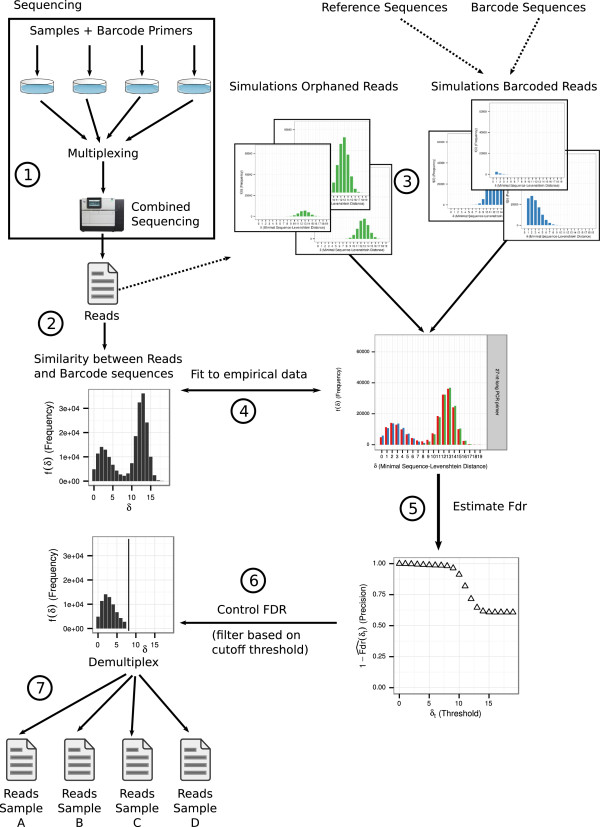
**Overview of the approach.** **(1)** A multiplexed sequencing experiment is conducted on the PacBio SMRT platform **(2)** The similarity between the obtained reads and the used barcode sequences is calculated. We show it as a histogram of distances. **(3)** We simulate orphaned reads and barcoded reads. The input to the orphaned reads simulation are fragments of the empirical reads. Input to the barcoded reads simulation are known barcode sequences attached to reference sequences. **(4)** Simulations are repeated for different parameter combinations. We modify parameters until the simulated data closely matches the empirical data. **(5)** The false discovery rate is estimated from the proportions of barcoded and orphaned reads for each possible distance value. **(6)** A satisfying false discovery rate (e.g., 0.05) is used to choose a threshold for the highest acceptable dissimilarity between reads and the barcode sequences. All reads with a higher distance to the used barcodes are discarded. **(7)** Reads are matched with their original samples (de-multiplexing).

Lastly, we validate the method using real multiplexed DNA sequencing data obtained on the PacBio SMRT platform, which includes the detection of barcoded reads, assigning barcoded reads to their samples and finding sequence variants.Figure 1 depicts an overview of the approach in practice.

## Methods

### Barcode preparation

The Sequence-Levenshtein distance between two DNA sequences A and B is the *minimal* number of insertions, deletions, and substitutions necessary to transform one sequence into any prefix of the other or vice versa. This property makes it suitable for use in DNA context (for full definitions and descriptions of this distance and algorithms described in this subsection see our previous work, [7]). The prefix of a sequence can be an empty sequence, the sequence itself, or any subsequence starting from position 1 up to any end position. A fast dynamic algorithm was available for the calculation of the Sequence-Levenshtein distance *d*_*SL*_(*A*,*B*) between any two sequences *A* and *B* (we make the algorithm available in Additional file 1).

The minimum Sequence-Levenshtein distance *δ* between a *set* of barcodes *BC* and a sequence *s* is the minimum of the distances between the barcodes and the sequence:


Barcode sets were built to ensure the Sequence-Levenshtein distance between every pair of barcodes to be at least . Such a barcode set allows the correction of at least 1 insertion, deletion, or substitution in DNA context. For the experiment, a set of 20 7-nt-long DNA multiplexing barcodes based on the Sequence-Levenshtein distance was prepared (list of barcodes in Additional file 2, Section S2).

We decided not to use PacBio’s original set of 16-nt-long DNA multiplexing barcodes for the following reason. In previous work, we have shown that more errors accumulate in longer sequences. Furthermore, we did not find information whether PacBio-barcodes were systematically engineered to optimise their robustness under such adverse conditions. Thus, we decided to use our own barcode design [7]. It was specifically designed to optimize the robustness and length of sequences for a given number of samples.

In the following, we will identify barcode sets by the length of their barcodes and the minimal Sequence-Levenshtein distance between them. We denominate a set with barcodes of length *l* and minimal Sequence-Levenshtein distance *d* as “[l,d] barcode set”. Hence, the aforementioned experimental barcode set will be called “[7,3] barcode set”. For simulation purposes, other barcode sets of various set size or barcode length (e.g., 20 × [6,3], 150 × [8,3], various [12,3] etc.) were generated using the same algorithm.

The barcode sets were generated heuristically: First, we generated an initial set of eligible fixed-length DNA sequences excluding those with a GC-content of less than 40% or more than 60%, perfect self-complementation, or more than two sequential repetitions of the same base. Second, from the eligible DNA sequences we randomly chose a set of three sequences with a pairwise Sequence-Levenshtein distance of at least three (the *seed*). Third, we scanned DNA sequences in lexicographic order and added a sequence to the seed if the newly added DNA sequence had a Sequence-Levenshtein distance of 3 from each sequence already in the set [23]. We repeated the third step for a large number of iterations with different random and randomly modified seeds [24].

When necessary for comprehension, we will denominate known barcodes as “*barcode reference sequences*” or “*reference barcodes*”. In our terminology, sets of barcodes are without exception sets of reference barcodes.

### Similarity of barcodes and barcoded primers to unbarcoded mRNA and DNA

The reference genome of *Mus musculus*, reference mRNA for all *M.musculus* transcripts, and the reference sequence of the murine *Atp1a1* transcript of the gene for Na+/K+-ATPase (NM_144900.2) were acquired from NCBI [25–27]. For similarity simulations, we sampled 10 million random 50-nt-long subsequences from the reference transcripts.

Similarities are tested between the aforementioned set of subsequences and sets of barcodes or so-called *barcoded PCR primer sequences*. The latter are concatenations of reference barcodes and primer sequences that were used to amplify the Atp1a1 transcript in our experimental validation (The experiment is described in Section “Experimental validation”. The barcodes and barcoded PCR primers are listed in Additional file 2, Sections S2 and S3). The degree of similarity between these barcodes or barcoded PCR primers and sampled subsequences was established by counting the frequency of their minimum Sequence-Levenshtein distances *δ* (see Equation 1).

Formally, the frequency function *f*(*δ*) of the minimal Sequence-Levenshtein distances *δ* between a set *S* of sequences and a set *BC* of barcodes or barcoded PCR primers was defined as:
1

(The frequency of the minimal Sequence-Levenshtein distance *δ* is the number of sequences *s* that have such a minimal Sequence-Levenshtein distance with the set *BC*).

The cumulative distribution function of *f*(*δ*) was defined as:


### Simulation of barcoded and orphaned PacBio reads

We begin with a set of experimental reads *S*^*emp*^ which we want to simulate for further analysis (c.f., Figure 1, we explain the generation of such a set *S*^*emp*^ in detail in the experimental section “Experimental validation”).

The set *S*^*sim*^ of simulated reads is a union between a set  of barcoded reads and a set  of orphaned reads. The purpose of the simulated read set *S*^*sim*^ is to closely resemble the properties of the targeted set of the empirical reads *S*^*emp*^ in regards to the minimal distances *δ* between the reads and the respective reference barcode set *BC*.

Simulations of reads must to be individually adapted to the particular simulated technology. Here, we target PacBio Continuous Long Reads (also called “subreads” and henceforth just *“reads*”) for which we developed our own read simulation. Our simulation assumptions rely on findings of Ono et al. [28]:

- accuracies of reads are normally distributed
- probabilities of sequencing errors per base are uniformly distributed over positions
- probabilities for insertions, deletions, and substitutions are possibly unequal
- differences in spatial distribution patterns of insertions, deletions, and substitutions are negligible

Hence, the following parameters governed the composition and traits of the read sets:

- The number *m* of reads in the set *S*^*sim*^
- The fraction *π*_1_ of reads that started with a barcode
- The average base sequencing error rate *μ*_*error*_
- The standard deviation of the base sequencing error rate *σ*_*error*_
- The ratios *R*={*R*_*INS*_,*R*_*DEL*_,*R*_*SUB*_} of insertions, deletions, and substitutions.

Set *S*^*sim*^ of all simulated reads was thus described as:


In the simulation and for a given set of parameters *m*,*π*_1_,*μ*_*error*_,*σ*_*error*_, and *R*, sets  and  were generated as follows:

For set , we constructed ⌊*π*_1_·*m*⌉ reads by choosing barcode reference sequences randomly from set *BC* and appending the reference sequence of the experimentally targeted insert. We then mutated the bases of each read randomly. The per-base sequencing error probability was  with *P*_*error*_ being fixed for each sequence. The respective probabilities of individual operations *OP*∈{*INS*,*DEL*,*SUB*} were then


Set  of orphaned reads was generated by choosing ⌊(1-*π*_1_)·*m*⌉ random 50-nt-long subsequences of the experimental reads *S*^*emp*^ starting after position 40. We chose this particular simulation set as we could reasonably assume these subsequences do not start with a barcode and have almost identical characteristics to the experimental orphaned reads.

### Frequency of test statistic in simulated data

The frequency distribution of such a set *S*^*sim*^ was the sum of the frequency distributions of both sets  and :


For the purpose of this method, we defined the frequency distribution of barcoded reads as the estimate of the alternative hypothesis distribution and the frequency distribution of orphaned reads as the estimate of the null hypothesis distribution, so that


and


The cumulative distribution functions were respectively


and


The estimated cumulative distribution function was then given by:


### Fitting simulated read sets to empirical read sets

In the next step, we fitted one set of simulated reads *S*^*sim*^ to the set of empirical reads *S*^*emp*^. Parameter *m* was predetermined by the number of reads in the empirical data set (*m*=|*S*^*emp*^|).

The parameter *R* of ratios between insertions, deletions, and substitutions had to be supplied by the user, for example, based on information supplied by the manufacturer or experimentally derived knowledge. We chose to estimate these ratios from those reads that had a very high likelihood of having been barcoded, identified as reads with a minimal distance of exactly *δ*=1 to the set of barcodes. For these reads we determined the sequencing errors that corrupted the barcoded reference primer sequence through alignment and calculated the ratios of sequencing error types.

To fit one set of simulated reads to the set of empirical reads, we used an evolutionary algorithm to search for the remaining parameter set (*π*_1_,*μ*_*error*_,*σ*_*error*_) that best explained the encountered frequency of similarities *f*^*emp*^ of the experimental sequences [29]. The fitness (i.e., the eligibility) of a particular parameter set was the root mean square (RMS) Euclidean distance between the simulated frequency distribution of minimal barcode distances *f*^*sim*^ and the experimental frequency distribution of barcode distances *f*^*emp*^:


The parameter set with the best observed fitness (i.e., lowest RMS) was selected to generate the simulated data set that was the closest to the empirical data set.

### Tail area-based false discovery rate

By adjusting the highest acceptable minimal distance *δ*_*t*_ (the so called *threshold*) between a set of reads and set of barcodes to distinguish between barcoded and orphaned reads, we manipulated (i.e., controlled) the false discovery rate of detecting barcoded reads. We defined the tail area-based false discovery rate (i.e., the fraction of barcode calls that are incorrect) as follows:


For the interpretation of biological results, we preferred to work with precision values (i.e., the fraction of barcode calls that are correct) which were given by:


Sensitivity (i.e., the fraction of sequences with actual barcodes that are correctly called) was then defined as:


### Precision and sensitivity of assigning reads to samples

Experimental reads are classified as starting with a barcode when their minimal barcode distance *δ* is equal to or below a chosen threshold distance *δ*_*t*_. We equivalently simulated this form of detection of barcoded reads by calculating an estimated set of barcoded reads  as a subset of all simulated reads *S*^*sim*^:


In this step, we decoded the (possibly altered) barcode that starts the read and assign the read to its original sample accordingly. When decoding only those reads in the set  and comparing the decoded barcodes with the reference barcodes used to generate set , we defined precision (i.e., the fraction of reads with detected barcodes that were correctly assigned to their original samples) and sensitivity (i.e., the fraction of all reads that were correctly assigned to their original samples) as:


### Experimental validation

To validate the Fdr approach, we asked whether we could successfully identify single-nucleotide variants (SNVs) within the genomic portion of samples that were sequenced in multiplexed fashion. C57BL/6 murine melanocytes were plated in 150 mm culture dishes at 10,000-40,000/*cm*^2^ and 18 hrs later they were exposed in PBS to 0, 500, 1000, 2000, or 3000 *J*/*m*^2^ of narrowband UVB radiation (principally 311±2*nm*; Philips, Eindhoven, Netherlands). After allowing 2-4 days for mutation expression (∼2 cell doublings), cells were incubated for 7 or 14 days in medium containing 10 mM ouabain octahydate (g-strophanthin, Sigma, St Louis, MO) to select for cells mutated in the Na+/K+-ATPase sodium pump [30, 31]. Clones larger than 100 cells were isolated and expanded. For 20 of the clones, total RNA was isolated, reverse transcribed to cDNA, and PCR amplified. Because UV mutation frequencies are ∼10^-4^ per gene, each clone is expected to have only 1 mutation (SNV) in the *Atp1a1* gene. A heterozygous mutation confers ouabain resistance, so the SNV is expected to be present in ∼50*%* of the mRNA material, with the remainder wildtype. PCR amplicons were sequenced by PacBio single-molecule sequencing as follows.

Twenty barcoded PCR primer pairs were synthesized for the murine *Atp1a1* gene. Each pair consisted of one 7-nt-long barcode 5^′^NNNNNNN 3^′^ (details can be found in Additional file 2, Section S3) followed by the 20-nt-long sequence 5^′^GGGAGCTGCTCTCTTCTCTT 3^′^ (forward primer) and the same 5^′^NNNNNNN 3^′^ followed by 5^′^TATAAACCTTGCCCGCTGTC 3^′^ (reverse primer).

Total RNA was isolated from cells (RNeasy Mini Kit, Qiangen, Valencia, CA), reverse transcribed to cDNA (SuperScript III First-Strand Synthesis, Invitrogen, Carlsbad, CA), and the 3.4 kb *Atp1a1* cDNA spanning the start and stop codons amplified by PCR (PrimeSTAR Max DNA Polymerase, TaKaRa, Kyoto, Japan) and gel purified without UV illumination. The pico green assay (Invitrogen) was used to mix equal DNA amounts from the 20 samples, and the mixture was ligated to Pacific Biosciences (Menlo Park, CA) SMRT adapters to create circular molecules for single-molecule sequencing in two SMRT cells using Continuous Long Read mode [13]. Raw reads were pre-processed by PacBio by cutting of raw reads at the sequencing adapters to generate so called subreads, henceforth just “reads”.

A complete and unaltered forward read would be the concatenation of a barcode, the forward primer, the *Atp1a1* transcript sequence, the reverse complement primer, and the reverse complement of the same barcode. In practice, reads typically begin internal to the *Atp1a1* transcript sequence.

For detection of barcoded reads, we assembled the set *S*^*emp*^ of all empirical reads as the union of the read sequences and their reverse complements, because a complete read was supposed to have the identical barcode both at the 5’ and 3’ end. The frequency of minimal Distances *δ* between *S*^*emp*^ and a set of reference barcodes or reference barcoded primers *BC* is named *f*^*emp*^ and calculated as described in subsection “Similarity of barcodes and barcoded primers to unbarcoded mRNA and DNA”.

Final assignment of reads to their respective samples was executed by finding the reference barcode with the minimal Sequence-Levenshtein distance to either the 5’ or 3’ end of the read, provided this distance was below or equal to the previously determined threshold *δ*_*t*_. If no such reference barcode was found or more than one reference barcode with such a minimal distance was found, the read was not assigned to any sample.

### Variant calling

The reads of the 20 samples were stripped of their barcodes and then aligned to the *Mus musculus* reference mRNA using the software package bwa-mem (version 0.7.8, [25, 32]). Variant calling was performed using the software package SAMtools (version 0.1.19, [33]). Parameter details are elaborated in Additional file 2, Section S4. Variants with a Phred quality score below 30 (p = 0.001), or less than 20 high-quality aligned reads (DP4) were filtered out.

## Results

### Coincidental barcode similarities in the reference *Mus musculus*DNA database

**Figure 2:**
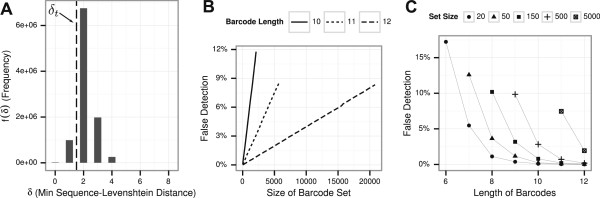
**Similarities between 10 million random subsequences of the** ***Mus musculus*** **DNA database and barcode sets of different sizes and barcode lengths.** **(A)** Distribution of minimal distances *f*(*δ*) between 150 [8,3] barcodes and subsequences. The separation by a naive threshold *δ*_*t*_=1 is illustrated by a vertical dashed line. **(B)** Falsely detected subsequences as proportion of all tested subsequences based on a threshold of *δ*_*t*_=*d*_*SL*_(barcode,subsequence)≤1 for different sizes of the barcode set and barcodes of length 10 nt, 11 nt and 12 nt **(C)** Falsely detected subsequences as proportion of all tested subsequences based on a threshold of *δ*_*t*_=*d*_*SL*_(barcode,subsequence)≤1 for different barcode lengths.

All our experimental and simulation barcode sets were designed to correct one insertion, deletion, or substitution error. However, DNA sequences of such length are frequently similar to naturally occurring *subsequences* of a *Mus musculus* genome. Figure 2(A) depicts the frequency of the similarity between a set of 150 [8,3]. Sequence Levenshtein barcodes and 10 million random 50-nt-long *subsequences* of the *Mus musculus* reference DNA database. In 1,080,761 cases one of the reference barcodes was equal to the reference subsequence or had a distance of only one (*d*_*SL*_(barcode,subsequence)≤1). Using this arbitrary threshold of *δ*_*t*_=1 (the error rate that the barcode set was designed to correct) to distinguish between barcoded and unbarcoded subsequences, we would have wrongly identified approximately 10.8% of subsequences as having been barcoded (which corresponds to 0% precision or a 100% Fdr). We will call this threshold *δ*_*t*_=1 the naive threshold and compare it to the Fdr method that we have developed.

Analyzing [12,3] barcode sets of different sizes (ranging from 20 barcodes to a maximum of 20,810 barcodes), we found a linear increase in proportion of subsequences that were falsely detected as barcoded based on the naive threshold of *δ*_*t*_=1. While only 428 subsequences (0.00428%) were falsely detected as barcoded when compared to the set of 20 barcodes, the ratio increased to 8.34% when the maximum set of 20,810 barcodes was tested (Figure 2(B)). The linear increase in proportion of falsely detected subsequences holds true for other barcode sets of 10 nt and 11 nt in length.

For a constant set size, the proportion of falsely detected subsequences decreased when the barcode length increased (Figure 2(C)). When using a set of 20 short [6,3] barcodes, 19.3% of subsequences were falsely detected to be barcoded, while this was true in approximately 0.004% of the subsequences when using the longer [12,3] barcode set that had the same number of elements. The relationship between barcode length and wrongly detected subsequences holds true for different barcode set sizes. If no information about inserts are available and only known barcode sequences are used for barcode detection, the results suggest to use at least 10-nt-long barcodes for 20 samples, 11-nt-long barcodes for 50 samples, 12-nt-long barcodes for 150 samples and even longer barcodes for larger sample sizes. It should be noted that longer barcodes come with problems of their own, as more mutations aggregate in longer barcodes. We will show in Section “Influence of attached reference PCR primer sequence on detection of barcoded reads” that a shorter barcode can be combined with knowledge about the insert template to alleviate the problems addressed in this section.

### Coincidental and genuine barcode similarities in *Atp1a1*sequencing data

In the experimental data, the expected complete size of the *Atp1a1* insert was 3,388 bp (including 7-nt-long barcodes at both ends). We collected 101,878 reads with an average length of 1,765 bp, and 95% of the reads were between 136 bp and 3,796 bp long. 89% of the reads were shorter than the expected complete coding sequence fragment and consequently many reads must have lacked a complete PCR primer and barcode at one or both ends. Reads longer than the targeted insert can also indicate other problems, for example a missed split at the SMRT adapter that may have led to absent or non-detectable barcodes.

#### Coincidental similarities in the *Atp1a1* genome

**Figure 3:**
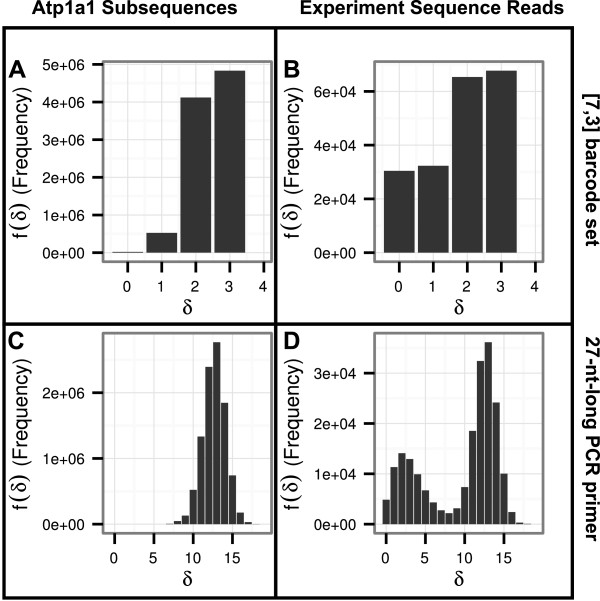
**Frequencies of minimal distances.** Frequencies of minimal distances from members of the [7,3] experimental reference barcode set or members of the 27 nt primer sequence set to randomly chosen subsequences of the unbarcoded gene *Atp1a1* (**A** and **C**, respectively), or minimal distances to experimentally observed reads of *Atp1a1* (**B** and **D**, providing *f* ^*emp*^(*δ*)).

Figure 3(A) shows the distribution of minimal distances between the unbarcoded transcript of *Atp1a1* and the experimental [7,3] reference barcode set. Correspondingly, Figure 3(C) depicts the distribution of frequencies between the unbarcoded transcript of *Atp1a1* and the set of complete barcoded 27-nt-long reference PCR primers.

As in the previous examples of coincidental barcode similarities, using the [7,3] barcode set and an arbitrary threshold distance of *δ*_*t*_=1 would have led to detecting barcodes incorrectly in approximately 6% of the subsequences (see Figure 3(A)). With the complete barcoded 27-nt-long reference PCR primer, similarities to subsequences of the *Atp1a1* transcript were much smaller and more seldom, with no subsequence having had a minimal distance of 3 or smaller to the set of barcoded PCR primers (Figure 3(C)).

#### Coincidental and real similarities in experimental *Atp1a1* reads

In the experimentally obtained *Atp1a1* sequence reads, at least a certain percentage of reads must have actually started with a barcode. We expect the reads to be a complex mix of correctly barcoded unaltered inserts, inserts with present but corrupted barcodes, and accidentally similar sequences. We repeated the previous similarity analysis with all experimental reads and their reverse complements, depicted as a histogram in Figure 3(B); and the minimal distance to each of the 40 27-nt-long barcoded PCR primers is shown in Figure 3(D).

As Figure 3(B) shows, there was no obvious visible separation value to distinguish barcoded from orphaned reads when using solely the [7,3] barcode set. Notably, the relative frequency of barcodes with no distance or a distance of 1 to the read was substantially higher than in Figure 3(A), yet it is unclear how many of the actual barcoded reads we could accurately detect by using a simple threshold value of *δ*_*t*_=0 or *δ*_*t*_=1.

In previous work, we have shown that in some situations it was possible to correct altered barcodes with a higher distance than the designated fault tolerance of the code (i.e., a distance higher than 1 in this particular [7,3] barcode set), because the average distance between reference barcodes was higher than 3 [7]. However, judging from the distribution depicted in Figure 3(A), a threshold of *δ*_*t*_=2 would have included too many orphaned reads: at least 49% of the reads would not have started with an actual (correct or altered) barcode and these reads would have been assigned to random clones, putting the variant calling step at risk.

In Figure 3(D), we depict the frequencies of minimal distances between the set of 27-nt-long barcoded reference PCR primers and the reads. A bi-modal distribution stands out, with one peak at a minimal Sequence Levenshtein distance *δ*=2 and another peak at a minimal Sequence Levenshtein distance of *δ*=13. The left peak is at approximately half the mean Sequence Levenshtein distance between every barcoded reference PCR primer of the used set (including PCR primer, *d*_*mean*_≈4.26,⌊*d*_*mean*_/2⌋=2) and the right peak is at approximately half the length of the barcoded reference PCR primer (*n*=27,⌊*n*/2⌋=13). The right peak is visibly consistent with the distribution in Figure 3(C), so we assumed this to be the distribution of the orphaned reads. The left distribution was accordingly assumed to be the distribution of correct and altered barcoded reads (i.e., barcoded reads). To summarize, a search for 7-nt-long reference barcodes in experimental sequencing data is problematic as no obvious separation could be found to distinguish barcoded and orphaned reads, even though such separation was clearly visible following a search for the barcode reference sequence with the attached PCR primer reference sequence. Defining a strategy to separate these two distributions for *both* the reference barcode set and the barcoded reference PCR primer set, and quantifying the quality of this separation was therefore the next important step.

### Detection of barcoded reads by Fdr

Figures 3(C) and (D) suggest that there is an extremely low likelihood to find *orphaned* reads that have a minimal distance of only 1 to one of the 27-nt-long barcoded reference PCR primers. We therefore used these particular reads to determine the ratio between insertions, deletions, and substitutions in this particular set of experimental data. We found that 55% of sequencing errors were insertions, 36.2% were deletions, and 8.8% were substitutions.

**Figure 4:**
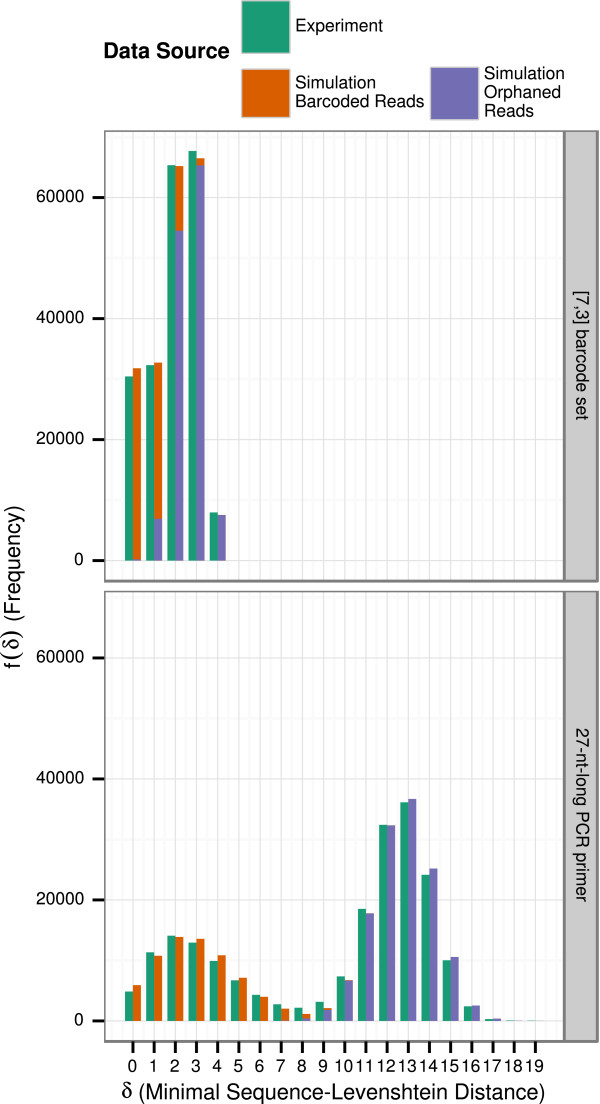
**Frequency distribution of similarities of experimental reads and simulated reads to the [7,3] barcode set and to the 27-nt-long barcoded PCR primer set.** The orange bars depict the frequency distribution of the minimal distances of the barcode or barcoded PCR primer set to the experimentally established reads *f* ^*emp*^(equal to Figure 3(B) resp. (D)). The slate blue and lime green bars depict the frequency distribution derived from a simulation, with the slate blue bar depicting the distribution of barcoded reads  and the lime green bar the distribution of orphaned reads . Bars of simulated frequencies were stacked.

Figure 4 depicts the distribution *f*^*sim*^(*δ*) of the simulation sequences *S*^*sim*^ fitted to distribution *f*^*emp*^(*δ*) of the empirical sequences *S*^*emp*^. The left distribution with a peak at *δ*=2 turned out to be the distribution of barcoded reads (), while the right distribution with a peak at *δ*=13 turned out to be the distribution of orphaned reads (). The obtained results were not noticeably different when using default ratios of insertions, deletions, and substitutions of 1/3, 1/3, 1/3 as opposed to using the empirically detected ratios. Nonetheless, to be as specific as possible we continued the remaining analysis with the empirically discovered ratios of errors.

We report that for this fit of simulated distributions to the empirical distribution the percentage of barcoded reads (*π*_1_) was approximately 34%. On average, *μ*_*error*_≈12.2*%* of the bases were altered by either an insertion, a deletion, or a substitution. This particular base sequencing error rate varied with a standard deviation of *σ*_*error*_≈4.8*%* between reads. The parameter solution was found reliably in every repetition of the simulation after a sufficient number of (usually 30-50) iterations to within a very small tolerance (<2 decimal digits). A common pitfall of evolutionary algorithms is the existence of “local solutions” (i.e., solutions with a monotonic score better than the immediate surrounding that is not the global solution). No such local solution was found in our simulations. The most resistant and reliable parameter found was the proportion of barcoded reads, as the form of the right distribution (peak at about *δ*=13) only depended on the proportion of barcoded reads (with the exception of extreme cases). Thus, the behaviour of the algorithm was very robust when applied on our data set.

**Figure 5:**
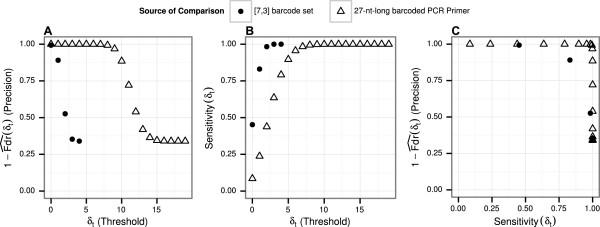
**Precision (1-** ***F*** ***d*** ***r*** **) and sensitivity of detection of barcodes in reads and their reverse complements.** Tests were conducted with the [7,3] reference barcode set (solid points) and the barcoded 27-nt-long reference PCR primers (empty triangles) **(A)** Precision of detection of barcoded reads for different thresholds **(B)** Sensitivity of detection of barcoded reads for different thresholds **(C)** Precision and sensitivity plotted against each other.

For this method, any read with a minimal Sequence-Levenshtein below or equal to a specific value (the *thresholdδ*_*t*_) was considered to be barcoded (see Methods “Tail area-based false discovery rate” for details). The simulation distribution allowed us to estimate the precision (1 - Fdr) and sensitivity of detecting barcoded reads in the experimental sequencing data (Figure 5) for every possible threshold value. This simulation was repeated using both the [7,3] reference barcode set and the set of 27-nt-long barcoded reference PCR primers.

For thresholding based on the [7,3] barcode set, the precision of detecting barcoded reads was very high (>99*%*) at the threshold of *δ*_*t*_=0 (exact match), while sensitivity of detection of barcoded reads stood at 46%. The use of such a threshold would have been ill-advised, as the purpose of using the error-correcting barcode was to correct at least one insertion, deletion, or substitution. At a threshold of *δ*_*t*_=2 detection precision fell below 53%. The compromise threshold of *δ*_*t*_=1 put the sensitivity of detection at approximately 82%, with a precision of 89%.

Using the complete barcoded 27-nt-long PCR primer reference sequence instead of the barcode reference sequence increased the quality of barcode detection substantially. The usage of the 27-nt-long barcoded PCR primer allowed a higher precision at equal sensitivity and reached a higher sensitivity at equal precision compared to using the [7,3] barcode set. The distance threshold *δ*_*t*_=9 was the highest to have a precision of more than 95%. At this threshold, detection sensitivity surpassed 99%.

### Influence of attached reference PCR primer sequence on detection of barcoded reads

**Figure 6:**
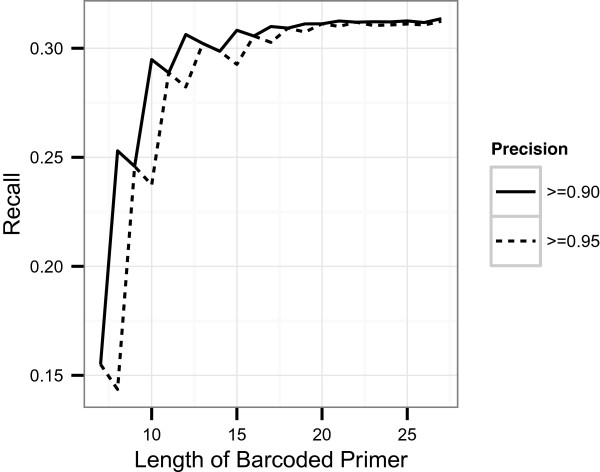
**Sensitivity of detecting reads with barcodes depending on length of barcoded reference primer.** Sensitivity was calculated for two different precision levels over barcoded reference primer lengths from 7 nt (just the reference barcode) to 27 nt (7 nt reference barcode plus 20 nt of reference primer appended). The staircase effect occurs due to discrete threshold steps and fixed precision levels.

Knowing that using the complete barcoded PCR primer reference sequence increased the quality of barcode retrieval, we tested detection of barcodes using concatenations of barcode reference sequences plus adjacent primer reference sequence fragments of different lengths, pictured in Figure 6. Sensitivity of assignment to experiments increased considerably with the use of the 17-nt-long barcoded reference PCR primer fragment (i.e., attaching 10 nt of the primer reference sequence to the reference barcodes) rather than only the 7-nt-long reference barcode set. Using longer barcoded reference PCR primer fragments increased the detection rate marginally, and it plateaued at approximately 20-nt-long barcoded reference PCR primers. As the computational cost did not prohibitively increase with increased lengths of the barcoded reference PCR primer sequence (computational complexity of calculating precision and sensitivity grows approximately quadratically over the length of the barcoded primer sequence), the full known barcoded primer sequence could be used. Although some implementation adaptions to the sequence simulation of Method 5 may be necessary for very long PCR primer sequences. In our particular case, a 20-nt-long barcoded PCR primer would have been sufficient.

### Assigning barcoded reads to their original samples

Detecting barcoded reads is only the first step in the demultiplexing protocol. In the next step, barcodes are decoded (i.e., error-corrected to find the reference barcode from which the corrupted barcode originates) and reads are assigned to the correct original sample.

**Figure 7:**
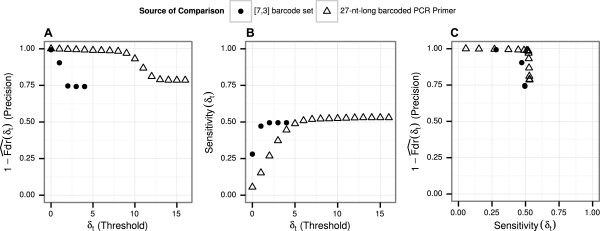
**Precision and sensitivity of assigning reads to samples.** Tests were conducted with the [7,3] barcode set (solid points) and the set of barcoded 27-nt-long PCR primers (empty triangles) **(A)** Precision of assigning reads to samples for different thresholds **(B)** Sensitivity of assigning reads to samples for different thresholds **(C)** Precision and sensitivity plotted against each other.

Figure 7 depicts the precision and sensitivity of this procedure. The highest sensitivity reached 53% when no thresholds were applied. Without a threshold, approximately 21.4% of the sequences were assigned to the wrong sample (Figure 7(C)). At a threshold of *δ*_*t*_=0 and when using the [7,3] barcode set, only 28% of reads could be assigned to their samples at a precision higher than 99%. For a threshold of *δ*_*t*_=1, sensitivity increased to 47% at a precision of 90%. The next higher threshold of *δ*_*t*_=2 saw precision drop to 74.6% with an increase in sensitivity to 49.5%. As in the previous analysis, the usage of the set of 27-nt-long barcoded reference PCR primers allowed a higher precision at equal sensitivity or reached a higher sensitivity at equal precision compared to using the [7,3] barcode set for assigning reads to samples (Figure 7(C)).

For the variant analysis of the experimental data, we decided on a threshold based on the set of barcoded 27-nt-long PCR primers that balanced high sensitivity with a high precision. We took into consideration that insufficient precision could have led to false variant calling, and insufficient sensitivity could have led to no variant calling at all. In the case of this experiment, sensitivity barely increased at a threshold higher than 7, and precision was very high (≥0.98). At that threshold, we could correctly assign 91% of reads that actually contained at least one barcode.

Of the 101,878 reads, we assigned 55,496 (≈54.5*%*) to their respective samples. An unambiguous assignment was not possible for 4,209 (≈4.1*%*) of the remaining reads which had different barcodes with the same minimal Sequence Levenshtein distance attached to the 5’ and 3’ end. The remaining 42,173 (≈41.4*%*) reads were classified as having no barcode at either end. Importantly, the median length of reads without any barcode was 1,248 nt, compared to a median length of 2,000 nt for those reads with at least one barcode at either end (see Figure S1 in Additional file 3). This supports the hypothesis that the former had genuinely no barcode at either end of the read. The median number of reads assigned per sample was 2,501.5 (mean 2,774.8, min 669, max 5,026).

### Variant calling

A search for *Atp1a1* sequence variants in the experimental data helped us to examine if our method was actually applicable to the real experimental design and how well it performed at avoiding cross-contaminating samples or reducing the number of usable sequence reads per sample. Detection of variants revealed five distinct *Single Nucleotide Variants* (*SNV*) in the gene *Atp1a1* in 18 of the 20 original samples and two further SNV in the remaining two samples (summarized in supplement Table S1 of Additional file 2). The former detected variants were consistent with the hypothesis of a *single* mutation in 50% of the mRNA material per sample. The SNV having two base changes one nucleotide apart, observed in two clones, is consistent with non-tandem double mutations occasionally caused by polymerase errors at and near a single DNA damage site after ultraviolet light.

Each variant call was supported by a large number of high-quality aligned reads, with coverage ranging from 184 to 195 (median 751.5 copies, mean 813.4 copies). The quality of variant calls was consistently high, with all Phred quality scores reaching 225. (Variant call files for *δ*_*t*_=9 are provided as Additional file 4 to this article.)

No changes in called variants were found when slightly lower or higher thresholds were tested (*δ*∈{7,8,10,11}). As Figure 7(A) shows, the 27-nt-long barcoded PCR primer is very resilient to small changes in the threshold. Still, a close examination of aligned reads assigned with different thresholds (using genome viewer IGV) showed signs of cross-contamination with reads that had a differing SNV. A screenshot is available in Additional file 5.

## Discussion

Multiplexed deep sequencing technologies are popular among researchers due to high information output and steadily decreasing processing time and costs. In multiplexing experiments, proper design of the barcodes is highly important. Careful consideration must be given to their physico-chemical and biological properties as well as to their error-correction capabilities. Here we demonstrated that using short barcode sequences exclusively is inefficient at assigning sequence reads to their respective DNA/RNA samples at high precision and sensitivity. Instead, additional information is needed such as the position of the barcode or adjacent primer sequences. Available deep sequencing platforms differ in their approaches to this problem. In Illumina HiSeq, for example, the genomic insert and barcode sequence are placed on different ends of an “index read primer” so that the sequence and the barcode are read separately [10, 11, 34]. However, this approach is not completely error-free. In addition, using positional information is not always possible either, since that technique is restricted to specific platforms and applications. If that technique is not available, a barcode is likely attached to the amplification/sequencing primer so that the primer sequence information can be used for barcode detection. Although this approach looks intuitively obvious, it is not clear what can be taken as the optimal solution for the choice of barcodes, primers, and detection algorithms. Additionally, sequencing errors add more noise to the data, which in turn requires proper thresholding for correct sequence assignment.

Our presented solution is built on the idea of controlling the tail area-based false discovery rate Fdr, and offers researchers a versatile tool to find an optimal threshold for detecting barcoded sequences. Additionally, it gives researchers a reliable impression of the quality of their threshold decision and the trade-off between precision and sensitivity, as well as facilitating further conclusions on the validity of the demultiplexing processing step. The method is generally usable for this particular problem, yet it needs to be modified to the specific technology and circumstances. The part of the method that needs to be adapted is the simulation of reads. Read simulation algorithms and analyses of read properties of common Next Generation Sequencing technologies can be found in the literature [35–37].

The approach of controlling the False discovery rate for a discrete test is new and still in an experimental stage. Nonetheless, recent development in the field of Fdr controlling procedures give the impression that exploiting the discreteness of the data increases reliability and sensitivity [38].

In this work, we focused on the specific advantages and issues of the PacBio SMRT platform, a next generation technology specialized in sequencing single large molecules [13]. Our protocol preferred sequencing primers attached to both ends of the DNA target. In this setup, barcodes can be easily added to the 5’-end of the PCR primers (DNA can be amplified before sequencing) so that a complete read has two identical barcodes from each sequencing end. In reality, for several reasons actual reads are quite infrequent in the expected form. One out of two barcodes is frequently missing. Technologically, with PacBio SMRT, the extension of the sequence by the immobilized polymerase and the reading may not be well synchronized. If the polymerase has been too fast or the deliberate time delay too long, the start of the insert could have been missed together with the barcode and the PCR primer. In some cases, the polymerase does not continue the reaction all the way to the end of the sequence. This means that the reverse complemented barcode at the end of the sequence may be missing as well [14, 15]. Finally, occasionally irrelevant mRNA/DNA fragments can be amplified during the PCR which allows further irrelevant reads without any barcodes to occur.

Having calculated similarities between barcodes or barcoded primers to the *Mus musculus* reference genome database, we see that longer barcode sequences generally show less randomly occurring similarities. This advantage is derogated by the number of barcodes used in the experiment: More barcodes increase the likelihood of coincidental similarities. The solution to this problem is to use longer barcodes or to concatenate barcodes with adjacent primer sequences.

Here we demonstrate the major dilemma of the optimality of the barcode design and identification. On the one hand, barcode sequences should be short and distinct to minimize different kinds of sequencing errors. On the other hand, a short barcode sequence is not unique in a genomic context and requires additional information for correct identification. For example, the barcode sequence itself can be extended by adding an adjacent primer sequence. This minimizes the false discovery rate due to decreased risk of coincidental similarities.

In this work, we found that using additional information from the PCR primer sequence improved barcode recovery tremendously. In future work, the experiment should be designed to handle the case where no such information is available. Firstly, adding an identical artificial sequence (a so called *stop-word*) to each barcode sequence solves the problem presented by redundancy of the words in big genomes. The best choice of stop-words is based on its dissimilarity to the targeted genome or insert. Furthermore, it is conceivable to generate Sequence-Levenshtein distance-based barcodes that, in combination with a known stop-word, creates an increased mean distance, which further increases the barcode set’s error-resilience. Secondly, sets of longer barcodes with error-correction capabilities beyond one error can be generated, which are beneficial to the overall statistics of the true barcode recovery.

The Fdr has to be calculated once per experimental data set, which includes the the simulation of reads and matching them to the experimental data. Computational complexity of the method grows quadratically over the length of the used barcode or barcoded primers. We found that longer barcoded primers increase sensitivity compared to shorter barcoded primers, while computational time was moderate in all cases. Additionally, we found that the increase in sensitivity plateaued for very long barcoded primers. Therefore, we believe that using a moderately long barcoded primer (≈20nt at 20 barcodes) offers the best reachable sensitivity performance and will still be computational feasible.

The statistical approach described here provides a solid method for finding an optimal threshold to separate barcoded and orphaned reads in real sequencing data sets. In addition to our main theme, the sample assignment of the genetic material was sufficiently precise and sensitive to generate a large number of high-quality and well-aligned reads. Consequently, exactly one SNV in the majority of samples and two SNVs in the remaining samples were found. The structure of the results indicated very low cross-contamination of insert read assignments caused by incorrect barcode calls and high-quality calls due to the large number of aligned reads at the respective SNV position.

PacBio offers their own method for the detection of barcodes in circular consensus reads (*CCS*) as part of their Quiver analysis software [39]. It is based on scores generated by a Hidden Markov Model (*HMM*). Our method can be considered as an alternative approach to the same problem. In addition it offers additional benefits, such as a statistical insight in the reliablity of the decision in the context of hundreds of thousands of reads as well as the systematic discovery of an eligible threshold.

## Conclusion

We presented a method for enhancing the detection of barcoded reads that can be adapted to different sequencing technologies and protocols. The method is based on false discovery rate statistics that were designed to assess the likelihood of true positives in an ocean of coincidental positives. Based on the precision-sensitivity estimates derived with our method, individual users can decide on a proper cutoff (or threshold) to detect sequence reads as being barcoded. Users can quantify the quality of the assignment of reads to samples. Additionally, they can select their particular trade-off between precision and sensitivity, thereby increasing the confidence in the results even in highly error-prone situations. Depending on the outcome, performance of the method can be further improved by the use of longer barcodes with higher error-correcting properties, or elongating the barcode by utilizing adjacent adapter or PCR primer sequences during computational detection to increase sensitivity.

## Electronic supplementary material

Additional file 1: **Dynamic algorithm of sequence-Levenshtein distance.** A fast algorithm to calculate the Sequence-Levenshtein distance between sequences *A* and *B*. (PDF 77 KB)

Additional file 2: **Supplement.** The supplement contains an exact definition of the Sequence Levenshtein distance, the list of experimental barcodes and primers, software parameters, more examples of coincidental similarities between barcodes, primers, and random subsequences of the *Mus musculus* DNA database, tables of detected variants, and tables of precision/sensitivity results. (PDF 135 KB)

Additional file 3: **Distribution of read lengths.** The figure depicts the distribution of read lengths, grouped in regard to their status as being barcoded at neither, one, or both ends. (PDF 13 KB)

Additional file 4: **Variant calls.** This archive contains the variant calls in bcf file format as exported by samtools. (ZIP 6 MB)

Additional file 5: **Evidence of cross contamination.** This screenshot from the genome viewer IGV shows signs of cross contamination in the aligned reads when a small threshold, middle threshold, and very high threshold was used. The depicted sample “ACCAGAA” had an SNV at position 2704. The screenshot shows variants at position 675, which is an SNV that was reliably found in other samples. (PDF 521 KB)
