# Supplementary material for: Enhancing the detection of barcoded reads in high throughput DNA sequencing data by controlling the false discovery rate
Source: BMC Bioinformatics. 2014 Aug 7;15(1):264. doi: 10.1186/1471-2105-15-264 (PMC4133078; doi:10.1186/1471-2105-15-264)
Supplement: Supplementary file 1 — Additional file 1: Dynamic algorithm of sequence-Levenshtein distance. A fast algorithm to calculate the Sequence-Levenshtein distance between sequences A and B. (PDF 77 KB) [file 12859_2013_6528_MOESM1_ESM.pdf]

Dynamic Algorithm of Sequence-Levenshtein distance

Algorithm 1 Sequence-Levenshtein Distance

```
function SequenceLevenshteinDistance(Sequence_1, Sequence_2)
    l_1 ← length(Sequence_1)
    l_2 ← length(Sequence_2)
    declare distances : array[l_1 + 1][l_2 + 1]
    for i ← 0, l_1 do
        distances[i][0] ← i
    end for
    for j ← 0, l_2 do
        distances[0][j] ← j
    end for

    for i ← 1, l_1 do
        for j ← 1, l_2 do
            if s_1[i − 1] = s_2[j − 1] then
                cost ← 0
            else
                cost ← 1
            end if
            distances[i][j] ← minimum(
                ▷ Substitution
                distances[i − 1][j − 1] + cost,
                ▷ Insertion
                distances[i][j − 1] + 1,
                ▷ Deletion
                distances[i − 1][j] + 1)
        end for
    end for
    min_distance ← distances[l_1][l_2]

    ▷ Sequence-Levenshtein extension
    ▷ Truncation
    for i ← 0, l_1 do
        min_distance ← minimum(min_distance, distances[i][l_2])
    end for
    ▷ Elongation
    for j ← 0, l_2 do
        min_distance ← minimum(min_distance, distances[l_1][j])
    end for
    return min_distance
end function
```
