# Supplementary material for: Enhancing the detection of barcoded reads in high throughput DNA sequencing data by controlling the false discovery rate
Source: BMC Bioinformatics. 2014 Aug 7;15(1):264. doi: 10.1186/1471-2105-15-264 (PMC4133078; doi:10.1186/1471-2105-15-264)
Supplement: Supplementary file 2 — Additional file 2: Supplement. The supplement contains an exact definition of the Sequence Levenshtein distance, the list of experimental barcodes and primers, software parameters, more examples of coincidental similarities between barcodes, primers, and random subsequences of the Mus musculus DNA database, tables of detected variants, and tables of precision/sensitivity results. (PDF 135 KB) [file 12859_2013_6528_MOESM2_ESM.pdf]

Supplement

Section S1 is a verbatim excerpt of the Sequence-Levenshtein distance definition as outlined in our previous work [1].

S1 Sequence-Levenshtein Distance

We adapted the Levenshtein distances in such a way that the DNA context is taken into account and the length of the new mutated barcode in the sequence read is correctly identified. In the worst case, any barcode embedded in the sequence read will be surrounded by the sample sequence such that it decreases its distance to other sequences in the set.

The Sequence-Levenshtein distance between two arbitrary words A and B is the minimum number of the following three operations:

- Substitutions
- Deletions
- Insertions

which results in word $\bar{A}$, finalized by applying one of the following operations exactly once:

- Truncating $\bar{A}$ to match the length of B
- Elongating $\bar{A}$ to match the length and bases of B

The latter two operations do not increase the distance between A and B. It follows, that the distance between A and B is 0 if A is a prefix of B (and vice versa). For the purpose of this distance metric, we define in this case A to be equal to B.

The formal definition of our metric allowed us to prove that the Sequence-Levenshtein distance is indeed a "distance metric", so that codes based on this distance can correct k substitutions and indels in DNA context if their minimum distance is at least $d_{SL}^{min} = 2 * k + 1$.

S2 List of Experimental Barcodes

CCTGTAA	ACCGTTA	ATGCCTA
TTGCAGA	GGATTGT	CAAGTCA
GATCGAA	TACTGGA	CGGTATA
CTCTAGA	TCAAGGA	CCAACAA
GGAAGAA	GAAGCTT	CAGAGAA
CACCTAA	TAGCCAA	ACCAGAA
TGGAGTA	GCGATAA	

S3 List of Experimental Primers

Forward (Fw) and Reverse (Rw) experimental barcoded primers.

```
Fw CCTGTAAGGGAGCTGCTCTCTTCTCTT
Rw CCTGTAATATAAACCTTGCCCGCTGTC

Fw GATCGAAGGGAGCTGCTCTCTTCTCTT
Rw GATCGAATATAAACCTTGCCCGCTGTC

Fw TTGCAGAGGGAGCTGCTCTCTTCTCTT
Rw TTGCAGATATAAACCTTGCCCGCTGTC

Fw CTCTAGAGGGAGCTGCTCTCTTCTCTT
Rw CTCTAGATATAAACCTTGCCCGCTGTC
```

```
Fw GGAAGAAGGGAGCTGCTCTCTTCTCTT
Rw GGAAGAATATAAACCTTGCCCGCTGTC

Fw CACCTAAGGGAGCTGCTCTCTTCTCTT
Rw CACCTAATATAAACCTTGCCCGCTGTC

Fw TGGAGTAGGGAGCTGCTCTCTTCTCTT
Rw TGGAGTATATAAACCTTGCCCGCTGTC

Fw ACCGTTAGGGAGCTGCTCTCTTCTCTT
Rw ACCGTTATATAAACCTTGCCCGCTGTC

Fw GGATTGTGGGAGCTGCTCTCTTCTCTT
Rw GGATTGTTATAAACCTTGCCCGCTGTC

Fw TACTGGAGGGAGCTGCTCTCTTCTCTT
Rw TACTGGATATAAACCTTGCCCGCTGTC

Fw TCAAGGAGGGAGCTGCTCTCTTCTCTT
Rw TCAAGGATATAAACCTTGCCCGCTGTC

Fw GAAGCTTGGGAGCTGCTCTCTTCTCTT
Rw GAAGCTTTATAAACCTTGCCCGCTGTC

Fw TAGCCAAGGGAGCTGCTCTCTTCTCTT
Rw TAGCCAATATAAACCTTGCCCGCTGTC

Fw GCGATAAGGGAGCTGCTCTCTTCTCTT
Rw GCGATAATATAAACCTTGCCCGCTGTC

Fw ATGCCTAGGGAGCTGCTCTCTTCTCTT
Rw ATGCCTATATAAACCTTGCCCGCTGTC

Fw CAAGTCAGGGAGCTGCTCTCTTCTCTT
Rw CAAGTCATATAAACCTTGCCCGCTGTC

Fw CGGTATAGGGAGCTGCTCTCTTCTCTT
Rw CGGTATATATAAACCTTGCCCGCTGTC

Fw CCAACAAGGGAGCTGCTCTCTTCTCTT
Rw CCAACAATATAAACCTTGCCCGCTGTC

Fw CAGAGAAGGGAGCTGCTCTCTTCTCTT
Rw CAGAGAATATAAACCTTGCCCGCTGTC

Fw ACCAGAAGGGAGCTGCTCTCTTCTCTT
Rw ACCAGAATATAAACCTTGCCCGCTGTC
```

S4 Variant Calling Parameters

S4.1 BWA-SW

The call to bwa-mem [2] was as follows:

```
bwa mem -t 16 -B 2 -O 2 -E 1 refMrna.fa <sample.fasta> -f <sample.sam>
```

S4.2 Samtools

The call to samtools [3] was as follows:

```
samtools mpileup -E -d10000000 -uf refMrna.fa <sample.bam>
```

S5 Coincidental Similarities to Mus Musculus DNA Database

Figure 1 gives more examples of the frequency distributions of Figure 1 (A) in the main manuscript.

Figure 2 extends Figure 1 (C) of the main manuscript by depicting the proportion of subsequences that are detected to be barcoded based on different thresholds.

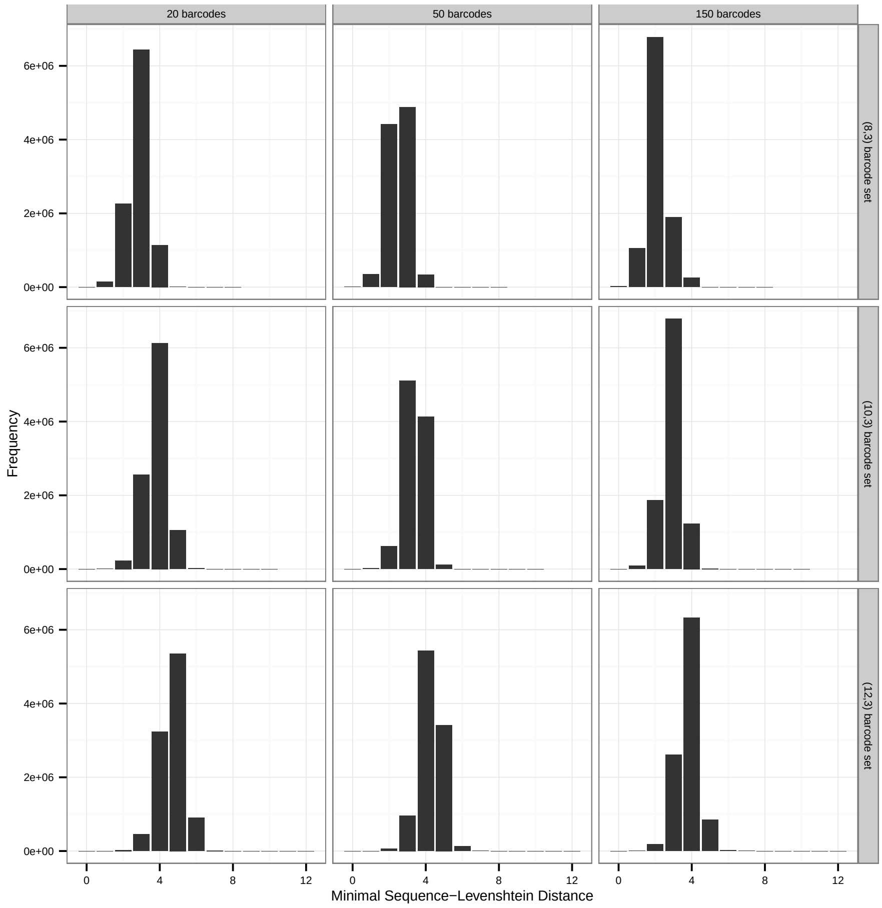

Figure 1: **Similarities between 10 million random subsequences of the Mus Musculus DNA Database and Barcode Sets of sizes 20, 50, and 150 and lengths 8nt, 10nt, and 12nt.** The figure expands on Figure 1 (A) of the main manuscript, providing more examples of frequency distributions.

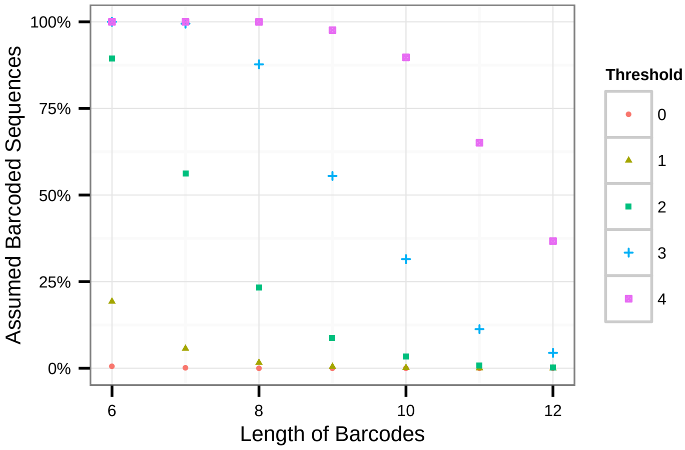

Figure 2: **Proportion of sequences detected as starting with a barcode, depending on length of the barcodes to which they are compared.** In a comparison of 1 Mio subsequences of the *Mus Musculus* DNA database with sets of 20 barcodes of different lengths, we found that longer barcodes show less incidences of coincidental similarities.

S6 Variant Call Results

Clone	Base	Mutation	Position	Phred Quality Score	DP4 sum
ACCAGAA	C	T	2704	225	768
ACCGTTA	G	C	675	225	1020
ATGCCTA	T	A	2674	225	717
ATGCCTA	A	T	2676	225	662
CAAGTCA	G	C	675	225	1244
CACCTAA	C	T	2704	225	720
CAGAGAA	C	T	2704	225	782
CCAACAA	C	T	2704	225	849
CCTGTAA	C	T	2704	225	1188
CGGTATA	G	C	675	225	549
CTCTAGA	A	G	676	225	1386
GAAGCTT	T	C	2692	225	593
GATCGAA	C	T	2704	225	966
GCGATAA	T	A	2674	225	195
GCGATAA	A	T	2676	225	184
GGAAGAA	G	A	675	225	735
GGATTGT	G	C	675	225	694
TACTGGA	C	T	2704	225	979
TAGCCAA	G	C	675	225	1585
TCAAGGA	G	C	675	225	804
TGGAGTA	C	T	2704	225	575
TTGCAGA	C	T	2704	225	700

Table S 1: Single Nucleotide Variants in Experimental Data

S7 Detection Tables

distance	fdr	recall	source
0	0.00613	0.456	[7,3] barcode set
1	0.10791	0.830	[7,3] barcode set
2	0.47512	0.982	[7,3] barcode set
3	0.64653	1.000	[7,3] barcode set
4	0.66007	1.000	[7,3] barcode set

distance	fdr	recall	source
0	$0.00e+00$	0.0862	27-nt-long barcoded PCR Primer
1	$0.00e+00$	0.2389	27-nt-long barcoded PCR Primer
2	$3.28e-05$	0.4400	27-nt-long barcoded PCR Primer
3	$4.55e-05$	0.6352	27-nt-long barcoded PCR Primer
4	$7.30e-05$	0.7915	27-nt-long barcoded PCR Primer
5	$1.29e-04$	0.8959	27-nt-long barcoded PCR Primer
6	$1.81e-04$	0.9558	27-nt-long barcoded PCR Primer
7	$7.78e-04$	0.9832	27-nt-long barcoded PCR Primer
8	$6.52e-03$	0.9945	27-nt-long barcoded PCR Primer
9	$3.18e-02$	0.9985	27-nt-long barcoded PCR Primer
10	$1.14e-01$	0.9996	27-nt-long barcoded PCR Primer
11	$2.79e-01$	0.9999	27-nt-long barcoded PCR Primer
12	$4.60e-01$	1.0000	27-nt-long barcoded PCR Primer
13	$5.81e-01$	1.0000	27-nt-long barcoded PCR Primer
14	$6.36e-01$	1.0000	27-nt-long barcoded PCR Primer
15	$6.55e-01$	1.0000	27-nt-long barcoded PCR Primer
16	$6.59e-01$	1.0000	27-nt-long barcoded PCR Primer
17	$6.60e-01$	1.0000	27-nt-long barcoded PCR Primer
18	$6.60e-01$	1.0000	27-nt-long barcoded PCR Primer
19	$6.60e-01$	1.0000	27-nt-long barcoded PCR Primer

S8 Sequence Assignment Tables

distance	recall	fdr	source
0	0.153	0.00607	[7,3] barcode set
1	0.279	0.11476	[7,3] barcode set
2	0.310	0.38913	[7,3] barcode set
3	0.312	0.43963	[7,3] barcode set
4	0.312	0.44016	[7,3] barcode set

distance	recall	fdr	source
0	0.0291	0.00000	27-nt-long barcoded PCR Primer
1	0.0814	0.00157	27-nt-long barcoded PCR Primer
2	0.1490	0.00381	27-nt-long barcoded PCR Primer
3	0.2114	0.00650	27-nt-long barcoded PCR Primer
4	0.2581	0.00934	27-nt-long barcoded PCR Primer
5	0.2873	0.01170	27-nt-long barcoded PCR Primer
6	0.3025	0.01322	27-nt-long barcoded PCR Primer
7	0.3088	0.01455	27-nt-long barcoded PCR Primer
8	0.3111	0.01918	27-nt-long barcoded PCR Primer
9	0.3121	0.03615	27-nt-long barcoded PCR Primer
10	0.3133	0.08761	27-nt-long barcoded PCR Primer
11	0.3153	0.17971	27-nt-long barcoded PCR Primer
12	0.3182	0.28458	27-nt-long barcoded PCR Primer
13	0.3208	0.35370	27-nt-long barcoded PCR Primer
14	0.3221	0.38250	27-nt-long barcoded PCR Primer
15	0.3225	0.39038	27-nt-long barcoded PCR Primer
16	0.3225	0.39165	27-nt-long barcoded PCR Primer
17	0.3225	0.39174	27-nt-long barcoded PCR Primer
18	0.3225	0.39176	27-nt-long barcoded PCR Primer
19	0.3225	0.39176	27-nt-long barcoded PCR Primer

S9 Read Assignment Tables

distance	fdr	recall	source
0	0.00596	0.281	[7,3] barcode set
1	0.09711	0.471	[7,3] barcode set
2	0.25506	0.493	[7,3] barcode set
3	0.25909	0.494	[7,3] barcode set
4	0.25909	0.494	[7,3] barcode set

distance	fdr	recall	source
0	0.00000	0.0542	27-nt-long barcoded PCR Primer
1	0.00214	0.1508	27-nt-long barcoded PCR Primer
2	0.00473	0.2667	27-nt-long barcoded PCR Primer
3	0.00699	0.3709	27-nt-long barcoded PCR Primer
4	0.00950	0.4452	27-nt-long barcoded PCR Primer
5	0.01156	0.4886	27-nt-long barcoded PCR Primer
6	0.01300	0.5098	27-nt-long barcoded PCR Primer
7	0.01390	0.5189	27-nt-long barcoded PCR Primer
8	0.01763	0.5219	27-nt-long barcoded PCR Primer
9	0.03048	0.5232	27-nt-long barcoded PCR Primer
10	0.06988	0.5245	27-nt-long barcoded PCR Primer
11	0.13271	0.5265	27-nt-long barcoded PCR Primer
12	0.18867	0.5287	27-nt-long barcoded PCR Primer
13	0.21047	0.5298	27-nt-long barcoded PCR Primer
14	0.21429	0.5299	27-nt-long barcoded PCR Primer
15	0.21456	0.5299	27-nt-long barcoded PCR Primer
16	0.21456	0.5299	27-nt-long barcoded PCR Primer

References

[1] Tilo Buschmann and Leonid V. Bystrykh. Levenshtein error-correcting barcodes for multiplexed dna sequencing. Unpublished, 2013.

[2] Heng Li and Richard Durbin. Fast and accurate long-read alignment with Burrows-Wheeler transform. *Bioinformatics*, 26(5):589–595, March 2010.

[3] Heng Li, Bob Handsaker, Alec Wysoker, Tim Fennell, Jue Ruan, Nils Homer, Gabor Marth, Goncalo Abecasis, and Richard Durbin. The sequence Alignment/Map format and SAMtools. *Bioinformatics*, 25(16):2078–2079, August 2009.
