# Supplementary figures and images for: Enhancing the detection of barcoded reads in high throughput DNA sequencing data by controlling the false discovery rate
Source: BMC Bioinformatics. 2014 Aug 7;15(1):264. doi: 10.1186/1471-2105-15-264 (PMC4133078; doi:10.1186/1471-2105-15-264)

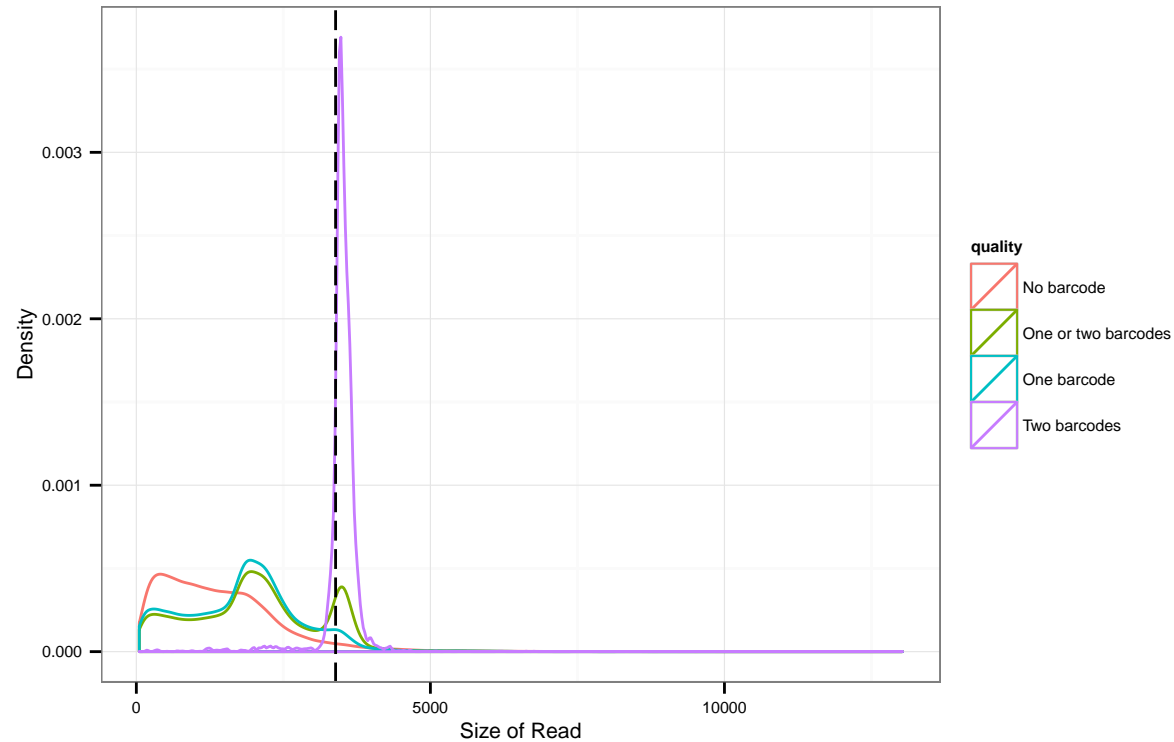

Supplement: Supplementary file 3 — Additional file 3: Distribution of read lengths. The figure depicts the distribution of read lengths, grouped in regard to their status as being barcoded at neither, one, or both ends. (PDF 13 KB) [file 12859_2013_6528_MOESM3_ESM.pdf]

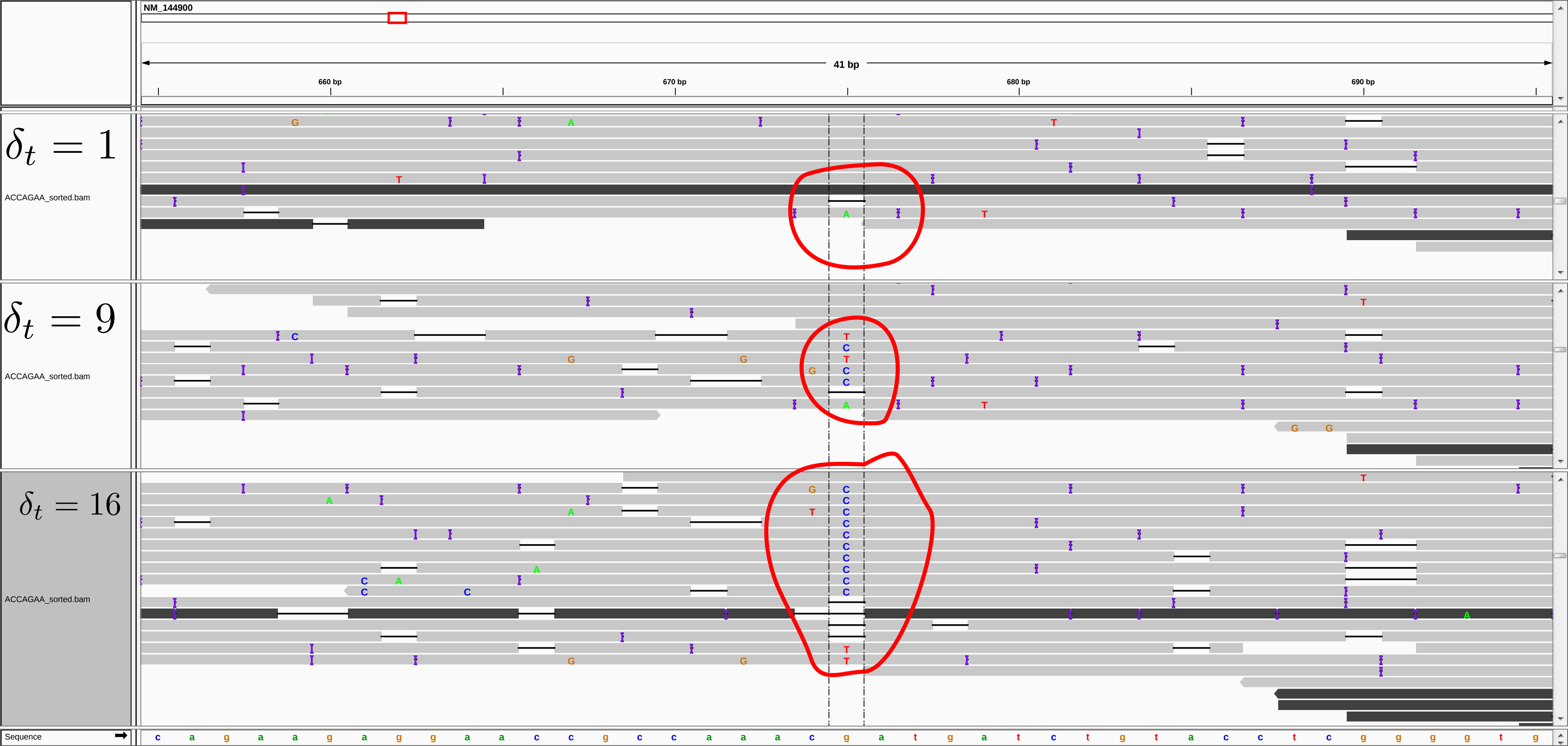

Supplement: Supplementary file 5 — Additional file 5: Evidence of cross contamination. This screenshot from the genome viewer IGV shows signs of cross contamination in the aligned reads when a small threshold, middle threshold, and very high threshold was used. The depicted sample “ACCAGAA” had an SNV at position 2704. The screenshot shows variants at position 675, which is an SNV that was reliably found in other samples. (PDF 521 KB) [file 12859_2013_6528_MOESM5_ESM.pdf]
